# Increase the Number of Views and Shares of COVID-19 Videos: Content Relevance and Emotional Consistency with Virus Variant Topics

**DOI:** 10.3390/healthcare11010119

**Published:** 2022-12-30

**Authors:** Jingfang Liu, Caiying Lu, Shuangjinhua Lu

**Affiliations:** School of Management, Shanghai University, Shanghai 200444, China

**Keywords:** COVID-19, video, uncertainty reduction theory, functional emotion theory

## Abstract

(1) Background: The coronavirus variants have posed serious challenges for the prevention and control of the COVID-19 pandemic. Individuals selectively watch and forward videos that help them reduce the damage caused by the virus. Therefore, the factors influencing video viewing and sharing in the context of the COVID-19 pandemic caused by virus variation must be explored. (2) Method: Based on a combination of uncertainty reduction theory and functional emotion theory, this paper designed hypotheses regarding how content relevance and emotional consistency affect video views and shares. We used the support vector machine (SVM) classification algorithm to measure the content relevance between videos and virus variant topics. We performed sentiment analysis of video text to evaluate the emotional consistency between videos and virus variant topics. Then, we used empirical analysis to build the model. (3) Results: The trained SVM classifier was effective in judging whether the video text was related to virus variant topics (F = 88.95%). The content relevance between COVID-19 videos and virus variant topics was generally low. The results showed that the higher the content relevance, the more views (IRR = 1.005, *p* = 0.017) and shares (IRR = 1.008, *p* = 0.009) the video received. Individuals were more willing to view (IRR = 1.625, *p* < 0.001) and share (IRR = 1.761, *p* < 0.001) COVID-19 videos with high emotional consistency with virus variant topics. (4) Conclusions: The results of empirical analysis showed that content relevance and emotional consistency between videos and virus variant topics significantly positively impacted video views and shares. The trained SVM classifier can support public health departments in monitoring and assessing the COVID-19 pandemic. Our study provides management advice while helping individuals reduce harm and inform next-step decisions.

## 1. Introduction

Since the COVID-19 pandemic spread around the world, the damage and changes to the global economy and trade, as well as people’s lifestyles and health, have been immeasurable [1,2,3]. The COVID-19 pandemic has attracted widespread attention. People have been fighting COVID-19 since its discovery in December 2019 [4,5]. As the situation improves, COVID-19 pandemic prevention and control have become normalized and must be balanced to ensure normal life can proceed [6].

COVID-19 belongs to the coronavirus family and has a single plus-stranded RNA genome. The unstable single-stranded structure means COVID-19 is prone to variation [7]. Variants of concern (VOC), named by the World Health Organization, are associated with increased transmissibility and harm, changes in clinical manifestations, public health management measures, existing diagnostic and therapeutic modalities, and reduced vaccine efficacy [8]. Virus variation is inevitable, predictable, and highly harmful. Therefore, the emergence of virus variation will pose new challenges to COVID-19 pandemic prevention and control.

Crisis refers to unexpected, sudden, and uncommon [9] events threatening human life and property [10]. In the context of the normalization of COVID-19 prevention and control, the sudden emergence of virus variants has produced unexpected and disruptive crises. Crises are characterized by uncertainty, suddenness, and destructive power. The timing of crises is unpredictable, so uncertainties exist in all stages of a crisis life cycle [11].

Internet platforms provide the easiest access to information, and more than 70% of adults use them when their health is at risk [12]. Information affects the effectiveness of individual knowledge acquisition and self-management on the Internet [13]. As an information carrier of Internet platforms, videos are deeply loved by contemporary Internet users and provide essential support for individuals to obtain information during a crisis [14]. During the COVID-19 pandemic, video-based information has become one of the most important information sources for the public [15,16,17], and studies on video information has also become meaningful.

Any form of interaction between individuals on Internet platforms during a crisis is meaningful [18] as interaction can reduce individual perceptions of uncertainty [19]. In the era of rapid Internet development, the public is accustomed to using Internet platforms to obtain information and interact with others [20,21]. Public access to crisis information through Internet platforms and the ability to share it with others can help individuals ease the effects of negative emotions caused by the crisis and determine their next steps [22]. When a crisis occurs, the public deepens their cognition of the crisis by collecting information, and individual behavior of obtaining information through videos involves watching videos. Information sharing can widely spread information and provides a method for individuals to communicate and interact with others. The information in videos is shared by forwarding videos [23]. During a crisis, the public watches and forwards videos to gain information and communicate with others to reduce their possibility of being harmed. Crises cause informational and emotional gaps [24]. Individual needs will change to reduce the gap, and people will forward and watch videos that can meet their informational and emotional needs.

In the context of the COVID-19 pandemic situation caused by the emergence of virus variants, in this study, we aimed to explore the impact of content relevance and emotional consistency between videos and virus variant topics on the number of views and shares of videos. We used the support vector machine (SVM) classification algorithm to calculate the content correlation between novel coronavirus videos and virus variant topics and then used sentiment analysis to calculate the emotional consistency between COVID-19 videos and virus variant topics. In this study, we included the calculated content relevance and emotional consistency scores in the independent variable index, and we conducted empirical to test the study hypothesis.

## 2. Theoretical Background

### 2.1. Literature Review

Many researchers have focused on the COVID-19 pandemic. The information factors that have been mined include information content [15], information quality [16], information sources [25], video language [26], etc. Regarding information content, scholars have used content analysis and natural language processing to classify the videos related to the COVID-19 topic. Szmuda (2020) divided COVID-19 videos into three categories through content analysis and found that, compared with videos related to historical pandemic and the zoonotic viruses, videos related to disease prevention received more views and were shared more often [15]. At present, the COVID-19 pandemic has been ongoing for almost 3 years. Individuals have gained a specific understanding of COVID-19 and have lost interest in watching and forwarding introductory popular science COVID-19 videos. Their demand has shifted to information they still need to learn, such as information related to new crises during the COVID-19 pandemic. Therefore, the content relevance between video and virus variants is an unstudied and may be a valuable information factor. In the context of the crisis caused by the COVID-19 variations, this paper explored the impact of the content relevance between videos and virus variant crises on the number of video views and shares.

The studies on emotion during the COVID-19 pandemic have mainly been conducted within the field of public opinion and have rarely considered the number of views and shares of videos. Researchers have focused on understanding public attitudes by analyzing public responses and interactive texts with the public health sector and news media. Huyen (2021) explored the public’s concern about the COVID-19 pandemic in Vietnam on different Internet platforms and found that the public’s attitude toward COVID-19 pandemic information changed from negative to positive as the COVID-19 pandemic progressed [26]. In addition, some researchers analyzed the emotions in public discussion texts to understand people’s response to the COVID-19 pandemic and conducted public opinion testing [27]. Maria (2021) compared the public’s information creation texts during the day and at night during a city’s lockdown and found that the public’s activities significantly increased at night, and the content turned to negative topics such as death [28]. The public expresses their opinions and emotions through texts. The emotions in texts are an external expression of the public’s internal emotional perception related to individual resonance with the external environment. When the emotion in a video is consistent with the environment, the public experiences emotional resonance with the video. These videos form a specific internal emotional perception, which affects the public’s behavioral intention in the crisis. Therefore, this paper incorporated emotional consistency as an independent variable when constructing factors influencing video views and shares.

### 2.2. Uncertainty Reduction Theory (URT)

Social psychology scholars think that only when people feel sure about their environment can they feel confident in their following actions and have reasonable expectations for the future [29]. Uncertainty is associated with reduced control [29], resulting in negative emotions and leading to hate; individuals tend to reduce or eliminate uncertainty [30].

Uncertainty is a primary state experienced by humans; as such, uncertainty management is a fundamental activity [31]. URT, developed based on uncertainty, was initially used to explain how people manage uncertainty in the early stages of social relationship building [32]. Berger and Calabrese suggested that a high degree of uncertainty leads to an increase in information-seeking behavior; as the level of uncertainty decreases, information-seeking behavior decreases (Axiom 3) [33]. Based on this axiom, communication scholars have applied URT to study the motivational construction of communication theory [29]. In studies on the adoption of close contact identification and risk warning in the COVID-19 context, Andreas considered uncertainty reduction as the motivation for application adoption. Social influence, transparent communication, and trust can reduce uncertainty and facilitate the use of COVID-19 information-tracking apps [34].

Crises create a high degree of uncertainty. Because crises are unpredictable and irregular events, uncertainty exists at all stages of the life cycle of a crisis [11]. URT states individuals undertake a series of behaviors to reduce the uncertainty during a crisis [35]. When individuals are exposed to an unexpected crisis, being affected by the surge of environmental uncertainty and the surrounding emotional tension, individuals act to reduce uncertainty by collecting crisis-related information and sharing it with others [36]. Crisis-related information can help individuals understand the current state of the crisis and make its development and future more predictable [37].

Due to aversion to uncertainty, also known as ambiguity aversion, individuals reduce uncertainty in the environment by collecting accurate details and sharing them with others [38]. Although inaccurate information can also spread during a crisis, people can quickly identify and correct inaccurate information through information sharing. Correct information helps reduce event ambiguity [39] and helps individuals to make correct choices [40]. This gathering and sharing of information prevents the further spread of false information, thereby reducing environmental uncertainty. Therefore, collecting and sharing information is considered a manifestation of individual attempts to reduce uncertainty in a crisis, which is effective in a crisis.

### 2.3. Functional Emotion Theory (FET)

Emotion is an individual’s internal psychological state as different responses to events, agents, or objects [41]. FET states that actions responding to emotions are adaptive functions to cope with individual internal change [42]. In the process of FET development, researchers have focused on different emotional characteristics. Nevertheless, the basic principles of FET can be summarized into four aspects: (1) emotions are generated in events that are closely related to individuals; (2) emotions have inherent adaptive functions; (3) each emotion produces a specific state of readiness for action or action-oriented state, which evokes, maintains, and manages an individual’s cognitive or physical activities; (4) emotions organize and motivate individual behavior [43,44].

Based on the above theories, scholars have described the process of FET as follows: an individual perceives the environment or an event related to them, the individual produces a unique emotional experience, and specific emotions manage the individual to produce a specific state of readiness for action or action-oriented state; in the end, the specific emotions affect individual cognitive or action, with the purpose of behavior being consistent with the emotional target [45]. In a study on fear appeals, Jiyeon used functional emotion theory to explain how emotions motivate behaviors that seek to protect relevant information. Negative emotions, such as fear and anxiety, motivate individuals to selectively search and process information related to protective behaviors to manage threats [46]. From a comparison of the relationship between emotions and behaviors of preschoolers facing a challenge alone or with their mother, researchers found that happiness when facing a challenge with their mother was associated with a broader range of behaviors, and anger when facing a challenge alone was associated with a wider range of behaviors. Dennis used functional emotion theory to explain the differences in emotions and adaptive behaviors in different scenarios [47]. These studies have shown that individual behavior depends on the setting of emotions. The perception and setting of individual emotions are related to the stimulus of the external environment and events.

## 3. Research Hypotheses and Models

### 3.1. Content Relevance with Virus Variant Topics

In a crisis, information can help individuals to reduce ambiguity [36], distinguish the usefulness of options [39], understand a crisis, and predict the future development of a crisis [37]. Individuals rely on crisis-related information to understand the current state, future, and what to do next [9]. During the COVID-19 pandemic, the public focused on information related to COVID-19, such as prevention measures, and paid less attention to historical pandemic [15]. Because COVID-19 prevention measures can help individuals better understand how to reduce their exposure to COVID-19, and the experience of a past pandemic only has reference value, direct and adequate information is more meaningful to individuals.

Crises are constantly changing, and unexpected events are uncontrollable [10]. When accidents occur, people tend to selectively collect and share information due to the high levels of uncertainty [11]. As a new crisis emerges, individual efforts to reduce uncertainty turn to reducing the uncertainty caused by new contingencies, which is related to the required information. The same is true of the COVID-19 pandemic. When virus variation occurs, videos related to the virus variant become the object of individual viewing and forwarding.

**Hypothesis** **1 (H1):** *When the COVID-19 coronavirus variation causes a crisis, the more relevant the video content to the virus variant topics, the more likely the video to be viewed by more people.*

**Hypothesis** **2 (H2):** *When the COVID-19 coronavirus variation causes a crisis, the more relevant the video content to the virus variant topics, the more people were likely to forward the video.*

### 3.2. Emotional Consistency with Virus Variant Topics

Emotions are considered distinctive, transient, and targeted, triggering different adaptive behaviors in response to the event causing the emotion [48]. In the context of a target event, emotions are more easily awakened. Emotions related to events can affect individual motivation and ability to participate in information processing [45]. In terms of the COVID-19 pandemic, the use of the Internet can cause negative emotions such as fear and anxiety, which can prompt individuals to take action to reduce their health threats [49]. The same is true when a virus variant occurs in the context of the target event. The higher the emotional consistency between the video and virus variant topics, the more likely the emotional perception of the watching individual arises, which produces behaviors to reduce their harm in the crisis. These behaviors include watching videos to gather information and forwarding videos to share information and interact with other.

**Hypothesis** **3 (H3):** *When the COVID-19 coronavirus variation causes a crisis, the higher the emotional consistency between videos and the virus variant topics, the more likely the video will be viewed by more people.*

**Hypothesis** **4 (H4):** *When the COVID-19 coronavirus variation causes a crisis, the higher the emotional consistency between videos and virus variant topics, the more likely the video will be forwarded.*

### 3.3. Research Model

On video platforms, to motivate creators, platforms will hold various events from time to time, providing creators with video exposure and creation incentives. Videos posted by creators participating in activities will gain additional exposure, and individuals may choose to watch and forward the video to engage in the activity. To push high-quality content to users, video platforms have established some leaderboards, which attract traffic to the videos on the leaderboard in the top positions. The content accuracy and video information quality released by public health departments and news media are higher than those of ordinary video producers. Hence, individuals trust them more. Individuals may view and forward their videos without trust in public health departments and news media. Therefore, we hypothesized that whether the video is an activity, whether it is included on a leaderboard, and whether the source is public health departments and news media would interfere with our study. We considered these variables as control variables in the model.

The independent variables in this study included content relevance and emotional consistency. Content relevance referred to the proportion of sentences referring to virus variant topics in the effective sentences in the video. We considered emotional consistency as the difference between the video’s emotional score and virus variant topics. The dependent variables included the numbers of video views and shares, and the control variables included activity videos, being listed on a leaderboard, and video source. The definitions of the variables are provided in Table 1.

**Figure 1:**
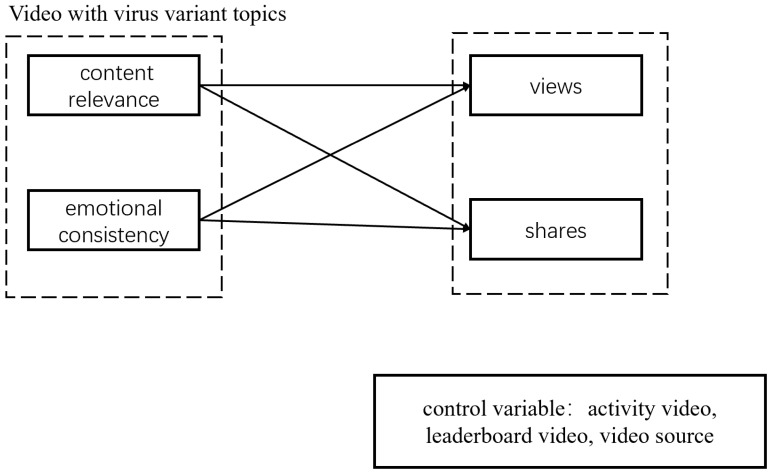
Research Model.

Figure 1 shows the research model of this paper.

## 4. Materials and Methods

**Figure 2:**
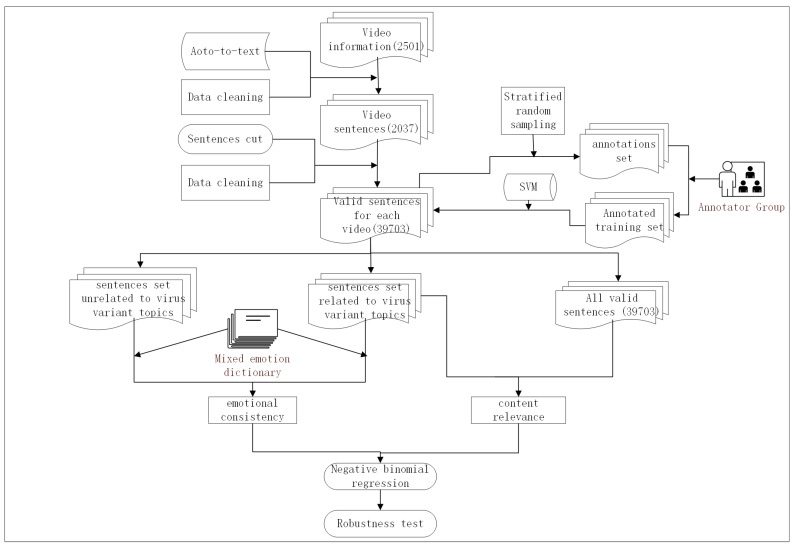
Research workflow.

During data preprocessing, we used the SVM algorithm to evaluate the content relevance between videos and virus variant topics, and we used sentiment analysis to evaluate the emotional consistency between videos and virus variant topics. For hypothesis testing, we used empirical analysis to test the impact of content relevance and emotional consistency between videos and virus variant topics on the number of video views and shares. The research workflow is shown in Figure 2, which we mainly divided into the following steps:

Step 1: Audio to text: We used mature audio-to-text technology to convert video audio into text.

Step 2: Sentence segmentation: We segmented the video audio that was processed into text, and we segmented the video audio text into several sentences.

Step 3: Data cleaning: We eliminated valid data should when the audio was converted to text and sentences were segmented.

Step 4: Conversion to text and sentence segmentation: With stratified random sampling and labeling, we randomly selected 4 sentences from each video as the labeling set. We invite researchers to label the sentences as related to virus variant topics or not, which formed the labeled data set.

Step 5: SVM classification: We used the annotated data set as the training set to train the SVM classifier with the linear kernel function, and all valid sentences were classified. The output of two data sets included a sentence set related to and a sentence set unrelated to virus variant topics. Then, according to the content relevance calculation formula, we calculated the content relevance between video and virus variant topics.

Step 6: Emotion analysis: We used the mixed emotion dictionary to analyze the emotion score of the two data sets, and we obtained the emotion score of each sentence and the emotion consistency score of each video.

Step 7: Negative binomial regression: We used the extracted independent variables (content relevance and emotion consistency) and dependent variables to perform negative binomial regression and hypothesis testing.

Step 8: Robustness test: We used the replacement variable measurement method to test the robustness of the model.

### 4.1. Data Sources

The research data in this paper comes from a well-known Chinese video platform, which is characterized by attention to originality and strong social functions. Originality ensures that videos on the platform are produced and published by the video owner. Videos on the platform rarely have different people publishing precisely the same content. Intense sociability indicates that users not only use the platform to obtain information but also are willing to socialize on the platform, such as by forwarding information. The platform and data characteristics met our study needs.

This paper used Chinese words related to virus variants and virus variant names such as “omicron” as search keywords to obtain video data. Our results of data analysis showed that May to August 2021 was the platform’s hottest time for virus variant topics. The number of videos published was above 500 per month, higher than the average of 238 in other months. In addition, the monthly growth rate in May 2021 was 25.37%, decreasing in August to 34.81%. Therefore, this article selected video data from May to August 2021, for a total of 4049 videos. The video data included the video screen and audio, video duration, views, shares, uploader certification type, activity video identification, leaderboard ranking, and other tags.

### 4.2. Data Preprocessing

Platform’s search mechanism finds keywords that appear in the video’s title, tag, description, or the publisher’s nickname, and then displays the search results. Therefore, our search results were not necessarily related to the virus variant crisis. Most videos were 2–16 min in length, and videos less than 2 min contained limited information. In a crisis, users’ negative emotions make needs for information acquisition or sharing urgent, so videos longer than 16 min do not conform to user habit of fragmentary information acquisition. Therefore, we eliminated videos that were entirely irrelevant to the COVID-19 topic through manual reading, and we removed videos that were shorter than 2 min and longer than 16 min, for a total of 2501 remaining videos.

This study used mature speech-to-text technology to convert the data of the 2501 videos from audio to text. The conversion accuracy was more than 99%, which met the study requirements. Through text processing, we found that some videos did not use human voice dubbing, so the audio-to-text result was empty. We removed the videos for which the result of audio-to-text was empty, the language was not Chinese, or the language was utterly illogical and rough. We finally obtained 2037 videos that met the study requirements.

A video 2–16 min in length contains multiple sentences. This article uses the Chinese ending words as the segmentation basis and divided the 2037 videos into several sentences. The video text contained the uploader’s opening and closing remarks, transitions between topics, and other content unrelated to the video content, which needed to be eliminated. The final total was 39,703 sentences.

### 4.3. Support Vector Machine (SVM) and Content Relevance

SVM is a machine learning algorithm based on statistics. It has substantial advantages in binary classification problems, is widely used in natural language processing, so was appropriate for our study [50]. This paper selected 4 sentences from each video as the training data, and we invited two researchers to label each text according to whether the topic was related to virus variants. The labeling results were verified as reliable after inspection.

This paper used the “Jieba” library for word segmentation, processing the words after eliminating useless sentences. We used the disabled dictionary to remove meaningless words, imported the custom dictionary to increase the accuracy of the segmentation result, and replaced synonyms for words with the same meaning. Due to the limited information expressed by a single character, we retained only phrases with a character length greater than 2 in word segmentation. Then, we used TF-IDF to process the word vectors of the segmented data. The SVM using a linear kernel learned whether sentences were classifiers for virus variant topics. The accuracy of the SVM classifier was 89.37%, and the F1 value was 88.95%, indicating that the training was effective.

We obtained the data used in training the classifier from 39,703 sentences after segmentation, and each video had data included in the classifier. Therefore, our classifier could be appropriately applied in our experiments. Next, we used the trained classifier to analyze all 39,703 data items to determine whether they were related to virus variant topics.

The training classifier determined whether each sentence was related to virus variant topics, so that we could judge the correlation between the video and virus variant topics. The number of sentences related to virus variants indicated how many sentences related to virus variant topics were in a video. The total number of sentences indicated

(1) $$\begin{array}{ccc} content\;relevance & = & \frac{number\;of\;sentences\;related\;to\;virus\;variant\;topics}{total\;number\;of\;sentences}\times 100 \end{array}$$

### 4.4. Emotional Consistency

Sentiment analysis is a text analysis method used to explain emotion intensity [51]. We adopted a sentiment lexicon as the tool for sentiment analysis. The mixed sentiment lexicon can make a lexicon more complete. A self-built sentiment lexicon can be used to modify the weight of some words and add new words to be more suitable for the study context. This paper assigned an emotional score to each sentence after the video was segmented and used the emotional score to calculate the emotional score of the video. Through the emotional score of the video, we could understand the emotional intensity of a video. The absolute value of the calculated video emotional score ranged from 0 to 6. The mean emotional score of sentences related to virus variant topics indicated the emotional intensity of the virus variant topic.

(2) $$\begin{array}{c} emotional\;consistency=6-\left|video\;sentiment\;score-virus\;variant\;topic\;sentiment\;average\;score\right| \end{array}$$

## 5. Results

### 5.1. Descriptive Statistical Analysis

We performed the empirical analysis in this study with Stata 16.0. The descriptive statistical analysis results of the 2037 video data are shown in Table 2. In terms of video emotion, the average emotional consistency score was 5.204, indicating that the video’s emotion on the platform was highly emotionally consistent with virus variant topics. The average content relevance score was 10.787, indicating that most videos covered more than just virus variant topics. The dependent variables (views and shares) were both non-negative integers. Their variance was much larger than the mean, so this paper used a negative binomial regression model for regression estimation.

### 5.2. Correlation Analysis

This paper used canonical correlation analysis to test whether the independent variables were strongly correlated, leading to multicollinearity. The results of the canonical correlation analysis (Table 3) showed that the correlation coefficients between the variables were below 0.3.

In addition, this paper evaluated the variance inflation factor of the independent variables. The results showed (Table 4) that the most significant variance inflation factor was 1.07, which is lower than the recommended threshold level of 10, indicating that multicollinearity was not an issue in this study [52].

### 5.3. Hypothesis Testing

In this study, we incorporated the independent variables (emotional consistency and content relevance) and control variables (activity videos, listed on leaderboard, and certification types) into the regression model. The dependent variables were the numbers of views and shares. The hypothesis test results of negative binomial regression are shown in Table 5.

H1 stated that the more relevant the video content to virus variant topics, the more views the video would receive. Model 1 in the table shows that when the content relevance score increased by one unit, the number of views increased by 0.5%, which was statistically significant (*p* = 0.017). So, H1 was supported. The results of the test of H2 proved that video shares (IRR = 1.008, *p* = 0.009) increased by 8% when the content relevance increased by one unit, and the effect was significant, supporting H2.

Model 1 also showed that when the video emotional consistency score increased by one unit, the number of views significantly increased by 52.5% (IRR = 1.625, *p* < 0.001), so H3 was supported as well.

We found from the test results of Model 2 regarding emotional consistency that with an increase in emotional consistency, video shares significantly increased (IRR = 1.761, *p* < 0.001), which supported H4.

### 5.4. Robustness Testing

This paper used punctuation marks as the standard when segmenting the sentences in the videos, where each segmented sentence expressed complete semantics. Therefore, we chose the number of sentences as the standard for calculating content relevance. Although sentences with shorter character lengths were eliminated, the character lengths of the sentences widely differed. In terms of video length, we found large differences in the proportions of each sentence. Therefore, this paper changed the method of calculating content relevance and used the character length of the sentence as the calculation standard to test the regression results and the robustness of the original model. We also proved that the model that replaced the method of calculating content relevance had no multicollinearity. The experimental results of negative binomial regression are shown in Table 6.

The model results after changing the method of calculating content relevance showed that the emotional consistency and content relevance between videos and virus variant topics significantly impacted video views and shares. The robustness test results supported the original model’s regression results.

## 6. Discussion

### 6.1. Main Conclusions

From the theoretical combination URT and FET, this paper used emotion analysis, the SVM algorithm, and empirical analysis to explore the factors influencing the number of video views and shares when COVID-19 variation caused tension among people.

First, functional emotion theory well explained the impact of emotional consistency on the number of views and shares in this study. This paper used the combination of URT and FET to explain the complexity of individual acquisition of information and sharing behaviors. FET could explain the impact of emotion on the number of video views and shares during the COVID-19 crisis. URT is a theory commonly used in the study of public health emergencies, but uses of FET to explain individual information collection and sharing behaviors in crises have been relatively rare. FET has often been used to understand the motivational effect of emotional perception on behavior. Previous studies focused on the influence of negative emotions on adopting protective behaviors. Nabi proposed discrete emotions as a psychological framework. In drunk driving scenarios, emotions stimulate relevant behavioral tendencies, which promote selective allocation of attention and information-seeking behaviors [53]. However, in different scenarios, the motivating emotion of a broader range of behaviors may be positive or negative, depending on the differences between the scenarios [47]. Therefore, from the perspective of the consistency between video and virus variant topics, this paper considered how COVID-19 videos caused the emotional settings related to the virus variant topics.

Second, this paper trained an SVM classifier to automatically divide the audio text into a set of sentences related to virus variant topics and a set of unrelated sentences. The trained SVM was effective, with an accuracy of 89.37% and an F1 of 88.95%. Previous researchers mostly used content analysis to classify video content [54], which required the watching of videos to manually mark the content, which limited the number of videos used. This paper extracted essential information from video employing audio-to-text conversion and used the SVM algorithm for automatic text recognition. The content relevance score indicated that videos were less relevant to the virus variant topics. A 2–16-min video can contain considerable amounts of information, discussing virus variations while conveying other information. A study of vaccine popularization information on YouTube also showed that a video can cover one or more topics in vaccine science and mechanism of action, vaccine trials, vaccine safety or side effects, and advocating preventive measures [55].

Third, the results of empirical analysis showed that the content relevance between videos and virus variant topics positively correlated with the number of video views and shares. Compared with historical pandemic video information, viewers are more likely to watch and forward videos related to COVID-19 [15]. Because historical pandemic information can only act as a reference, information about COVID-19 is more effective in reducing uncertainty by directly providing details. The information is not stable and consistent during a crisis [56]. The dynamic changes in information caused by the changes in critical information and arguments make the information consumption behavior full of uncertainty [57,58]. Only a few researchers have considered the characteristics of information-seeking and sharing behavior when the uncertainty of a crisis creates information demand. The infectivity, fatality rate, and incubation period of the novel coronavirus variants were all unknown. The environment in which an individual lives is full of uncertainties. Individuals are only interested in information that can address their current uncertainties. To reduce uncertainty, individuals have tended to seek information about virus variants. Individuals watch and forward videos that provide more relevant information, i.e., videos that are more relevant to the virus variants.

Fourth, our results showed that the higher the emotional consistency, the more a COVID-19 video is viewed and shared. Emotion, as the external manifestation of an individual’s feelings, is reflected in individual language and writing, which reflect the individual’s attitude toward events. In studies on emotion in crisis, researchers determined individuals’ emotional expression from individual language and writing to measure individual feedback on public crisis management [59]. However, the role of emotion as the internal force driving individual behavior in a crisis can be easily ignored. In the case of the tension caused by the COVID-19 variants, individuals’ information collection and sharing behaviors are related to emotions. Individuals tended to watch and forward videos on virus variant topics that conveyed emotion similar to theirs. When watching videos with high emotional consistency, individuals feel they are in an environment affected by virus variants, so are likely to generate corresponding emotional perceptions, such as fear and unease about virus variants. Such emotional perception prompts individuals to watch and forward the video.

COVID-19 prevention and control have always had to face the challenges produced by new crises, such as the emergence of new virus variants. From our findings, we provide several recommendations for public health departments, news media, and individuals concerned with the COVID-19 crisis.

First, the SVM algorithm classifier could identify whether the text in COVID-19 videos was related to virus variant topics. The coronavirus variations have led to crises during the COVID-19 pandemic, which have posed constant challenges to the prevention and control of the pandemic. The classifier could monitor the relevance between videos and virus variant topics and evaluate the change in viewer attention to virus variant topics. It can also provide technical support for popularizing highly relevant videos when a new COVID-19 variant emerges.

Second, this paper revealed that when the COVID-19 virus variant, the number of video views and shares is related to content relevance and the emotional consistency between the videos and virus variant topics. Specifically, the public is more willing to watch and forward videos highly related to virus variant topics and that have similar emotional expressions. Public health departments and news media use the Internet to provide information to the public and support for the public to use the Internet to collect and share information. This finding suggests that public health departments and news media should consider both the relevance of videos and virus variant topics, as well as whether the video’s emotion is close to the crisis emotion when providing video information to people in areas where the COVID-19 pandemic is serious.

Finally, for individuals in crisis, our findings explain what kind of video can help them understand the crisis and is suitable for sharing with others. In the case of unpredictable danger and the timing of crises, our findings can help individuals quickly find the information they need when a crisis occurs. Individuals can also reduce their uncertainty by watching and forwarding videos and feel sure about the next steps in a crisis.

### 6.2. Limitations of the Research

This study has many limitations. First, the sectional data we used could not reflect the changes over time in the number of video views and shares. The situations caused by the pandemic are variable, resulting in sudden tension, followed by relaxation as waves caused by the variants pass. The given situation considerably and quickly changes individual information-seeking and -sharing. Therefore, the time when a video is uploaded during a crisis is critical. In future studies, panel data of the number of video views and shares on specified days can be considered. The classification of time can also be more detailed. For example, when the information demand is high, we can use the classification of the video release time based on the search volume on Internet engines as the classification standard. Second, due to the limitation of the study methods and data, we did not consider the entertainment experience when individuals watched the videos. In addition to information and emotional needs, watching videos is a form of entertainment. Factors affecting individual viewing and immersion perception, such as the camera position of the video producer when recording the video, will also impact the number of video views and shares [60].

## 7. Conclusions

From the perspective of video content relevance and emotional consistency between videos and virus variant topics, in this study, we explored the factors that affected the number of video views and shares. The study leveraged the SVM classification algorithm to assess video content relevance and used sentiment analysis to provide support for scoring the sentiment coherence of the videos. The results showed that when the emergence of virus variants caused sudden tension during the COVID-19 pandemic, individuals tended to watch and forward videos whose content and emotional expression were similar to virus variant topics. Our findings can help public health departments better monitor the development of the COVID-19 pandemic and cooperate with news media to push information that more in line with the needs of the public. Our study can also help with providing timely, rapid, and accurate advice for individuals to respond to a new crisis, helping them confidently make decisions. The limitations in data and variable extraction in this study need to be addressed. In future studies, we will use panel data to improve consideration of time and other experimental methods to measure individual entertainment needs.

## Figures and Tables

**Table 1:** Variables definition.

Type	Name	Description
independent variable	content relevance	Content relevance score between videos and virus variant topics
	emotional consistency	Emotional consistency score between videos and virus variant topics
dependent variable	views	The number of views of videos
	shares	The number of shares of videos
control variable	activity video	Whether the video is an active work of the platform, 0 means it is not an active work, 1 means it is an active work
	leaderboard video	Whether the video is included in a certain ranking board of the platform, 0 means not included in any ranking board, Integers from 1 to 100 indicate a leaderboard ranking of 100-1
	video source	Whether the source of the video is public health departments and news media, 0 means the video comes not from public health departments or news media, 1 means the video comes from public health departments and news media

**Table 2:** Summary Statistics.

Variable	Obs	Mean	Std. Dev.	Min	Max
emotional consistency	2037	5.204	1.062	0.093	5.999
content relevance	2037	10.787	23.307	0	100
activity video	2037	0.407	0.491	0	1
leaderboard video	2037	0.105	2.379	0	62
video source	2037	0.369	0.483	0	1
shares	2037	218.166	1763.554	0	62,000
views	2037	55,013.988	190,142.38	2	3,384,454

**Table 3:** Pairwise Correlations.

Variables	(1)	(2)	(3)	(4)	(5)
(1) emotional consistency	1.000				
(2) content relevance	0.201	1.000			
(3) video source	0.197	0.148	1.000		
(4) activity video	0.053	0.102	0.092	1.000	
(5) leaderboard video	0.020	0.034	0.057	0.011	1.000

**Table 4:** Independent variable variance inflation factor.

Variable	VIF	1/VIF
emotional consistency	1.07	0.931
content relevance	1.06	0.940
activity video	1.02	0.983
leaderboard video	1.00	0.996
video source	1.06	0.941
Mean VIF	1.04	

**Table 5:** Hypothesis testing.

Variables	(1) Views			(2) Shares		
	IRR	*p*		IRR	*p*	
activity video	1.051	0.593		0.681	0.004 (***)	
leaderboard video	1.064	0.020 (**)		1.086	0.041 (**)	
video source	5.468	<0.001 (***)		5.062	<0.001 (***)	
emotional consistency	1.625	<0.001 (***)	H3	1.761	<0.001 (***)	H4
content relevance	1.005	0.017 (**)	H1	1.008	0.009 (***)	H2
_cons	1242.23	<0.001 (***)		1.121	<0.001 (***)	
lnalpha	1.417			2.026		
PseudoR2	0.012			0.023		
Observations	2037					

*** *p* < 0.01, ** *p* < 0.05.

**Table 6:** Robustness testing.

Variables	(1) Views			(2) Shares		
	IRR	*p*		IRR	*p*	
activity video	1.059	0.537		0.695	0.007 (***)	
leaderboard video	1.064	0.021 (**)		1.086	0.043 (**)	
video source	5.563	<0.001 (***)		5.196	<0.001 (***)	
emotional consistency	1.629	<0.001 (***)	H3	1.766	<0.001 (***)	H4
content relevance	1.004	0.056 (*)	H1	1.007	0.026 (**)	H2
_cons	1232.74	<0.001 (***)		3.227	<0.001 (***)	
lnalpha	1.418			2.028		
PseudoR2	0.012			0.022		
Observations	2037					

## Data Availability

Not applicable.

## References

[1] Wu F., Zhao S., Yu B., Chen Y.M., Wang W., Song Z.G., Hu Y., Tao Z.W., Tian J.H., Pei Y.Y. (2020). A new coronavirus associated with human respiratory disease in China. Nature.

[2] Shah S.G.S., Farrow A. (2020). A commentary on “World Health Organization declares global emergency: A review of the 2019 novel Coronavirus (COVID-19)” Comment. Int. J. Surg..

[3] Nicola M., Alsafi Z., Sohrabi C., Kerwan A., Al-Jabir A., Iosifidis C., Agha M., Agha R. (2020). The socio-economic implications of the coronavirus pandemic (COVID-19): A review. Int. J. Surg..

[4] Chen N., Zhou M., Dong X., Qu J., Gong F., Han Y., Qiu Y., Wang J., Liu Y., Wei Y. (2020). Epidemiological and clinical characteristics of 99 cases of 2019 novel coronavirus pneumonia in Wuhan, China: A descriptive study. Lancet.

[5] World Health Organization Pneumonia of Unknown Cause—China. https://www.who.int/csr/don/05-january-2020-pneumonia-of-unkown-cause-china/en/.

[6] Xue Y., Xu Q., Wang J., Lin H., Wang C., Lou X., Wu C., Mao Z., Fu X. (2022). Prevalence and Associated Factors for Elevated Depressive Symptoms in 386,924 Primary Students during the COVID-19 Pandemic Normalization in China. Int. J. Environ. Res. Public Health.

[7] Weiss S.R., Navas-Martin S. (2005). Coronavirus pathogenesis and the emerging pathogen severe acute respiratory syndrome coronavirus. Microbiol. Mol. Biol. Rev..

[8] Boehm E., Kronig I., Neher R.A., Eckerle I., Vetter P., Kaiser L. (2021). Novel SARS-CoV-2 variants: The pandemics within the pandemic. Clin. Microbiol. Infect..

[9] Seeger M.W., Sellnow T.L., Ulmer R.R. (2003). Communication and Organizational Crisis.

[10] Seeger M.W., Sellnow T.L., Ulmer R.R. (1998). Communication, organization, and crisis. Ann. Int. Commun. Assoc..

[11] Lee C.H., Yu H. (2020). The impact of language on retweeting during acute natural disasters: Uncertainty reduction and language expectancy perspectives. Ind. Manag. Data Syst..

[12] Fox S., Duggan M. Health Online 2013. https://www.pewresearch.org/internet/2013/01/15/health-online-2013/.

[13] Huang X., Fan J. (2022). Understand the Impact of Technology Feature in Online Health Communities: Why the Representation of Information Matters. Int. J. Hum.-Comput. Interact..

[14] Liu J.F., Lu C.Y., Lu S.J.H. (2021). Research on the Influencing Factors of Audience Popularity Level of COVID-19 Videos during the COVID-19 Pandemic. Healthcare.

[15] Szmuda T., Syed M.T., Singh A., Ali S., Özdemir C., Słoniewski P. (2020). YouTube as a source of patient information for Coronavirus Disease (COVID-19): Acontent-quality and audience engagement analysis. Rev. Med. Virol..

[16] Kocyigit B.F., Akaltun M.S., Sahin A.R. (2020). YouTube as a source of information on COVID-19 and rheumatic disease link. Clin. Rheumatol..

[17] Ataç Ö, Özalp Y.C., Kurnaz R., Güler O.M., İnamlık M., Hayran O. (2020). YouTube as an Information Source During the Coronavirus Disease (COVID-19) Pandemic: Evaluation of the Turkish and English Content. Cureus.

[18] Gascó M., Bayerl P.S., Denef S., Akhgar B. (2017). What do citizens communicate about during crises? Analyzing twitter use during the 2011 UK riots. Gov. Inf. Q..

[19] Lachlan K.A., Spence P.R., Lin X., Najarian K., Del Greco M. (2016). Social media and crisis management: CERC, search strategies, and Twitter content. Comput. Hum. Behav..

[20] Radonjic A., Hing N.N.F., Harlock J., Naji F. (2020). YouTube as a source of patient information for abdominal aortic aneurysms. J. Vasc. Surg..

[21] Madathil K.C., Rivera-Rodriguez A.J., Greenstein J.S., Gramopadhye A.K. (2015). Healthcare information on YouTube: A systematic review. Health Inform. J..

[22] Veil S.R., Buehner T., Palenchar M.J.A. (2011). Work-In-Process Literature Review: Incorporating Social Media in Risk and Crisis Communication. J. Contingencies Crisis Manag..

[23] Kwon J., Han I., Kim B. (2017). Effects of source influence and peer referrals on information diffusion in Twitter. Ind. Manag. Data Syst..

[24] Shklovski I., Palen L., Sutton J. The impact of language on retweeting during acute natural disasters: Uncertainty reduction and language expectancy perspectives. Proceedings of the 2008 Acm Conference on Computer Supported Cooperative Work.

[25] Li H.O.Y., Bailey A., Huynh D., Chan J. (2020). YouTube as a source of information on COVID-19: A pandemic of misinformation?. BMJ Glob. Health.

[26] Tran H.T.T., Lu S.H., Tran H.T.T., Van Nguyen B. (2020). Social Media Insights During the COVID-19 Pandemic: Infodemiology Study Using Big Data. JMIR Med. Inform..

[27] Ghanem A., Asaad C., Hafidi H., Moukafih Y., Guermah B., Sbihi N., Zakroum M., Ghogho M., Dairi M., Cherqaoui M. (2021). Real-Time Infoveillance of Moroccan Social Media Users’ Sentiments towards the COVID-19 Pandemic and Its Management. Int. J. Environ. Res. Public Health.

[28] Castaldo M., Venturini T., Frasca P., Gargiulo F. (2021). The rhythms of the night: Increase in online night activity and emotional resilience during the spring 2020 COVID-19 lockdown. EPJ Data Sci..

[29] Hogg M.A. (2011). Subjective uncertainty reduction through self-categorization: A motivational theory of social identity processes. Eur. Rev. Soc. Psychol..

[30] Fiske S.T., Taylor S.E. (1991). Social Cognition.

[31] Goldsmith D.J. (2001). A normative approach to the study of uncertainty and communication. J. Commun..

[32] Stefanone M.A., Hurley C.M., Yang Z.J. (2013). Antecedents of Online Information Seeking. Inf. Commun. Soc..

[33] Berger C.R., Calabrese R. (1975). Some explorations in initial interactions and beyond: Toward a developmental theory of interpersonal communication. Hum. Commun. Res..

[34] Oldeweme A., Märtins J., Westmattelmann D., Schewe G. (2021). The Role of Transparency, Trust, and Social Influence on Uncertainty Reduction in Times of Pandemics: Empirical Study on the Adoption of COVID-19 Tracing Apps. J. Med. Internet. Res..

[35] Berger C.R., Bradac J.J. (1982). Language and Social Knowledge.

[36] Berger C.R. (2007). Interpersonal processes: New directions for communication research. Communicating under Uncertainty.

[37] Neves P., Almeida P., Velez M.J. (2018). Reducing intentions to resist future change: Combined effects of commitment-based HR practices and ethical leadership. Hum. Resour. Manag..

[38] van den Bos K., Lind E.A. (2002). Uncertainty management by means of fairness judgments. Adv. Exp. Soc. Psychol..

[39] Farh C.I., Bartol K.M., Shapiro D.L., Shin J. (2010). Networking abroad: A process model of how expatriates form support ties to facilitate adjustment. Acad. Manag. Rev..

[40] Brashers D.E. (2001). Communication and uncertainty management. J. Commun..

[41] Ortony A., Clore G.L., Collins A. (1988). The cognitive structure of emotions. Communicating under Uncertainty.

[42] Darwin C.R. (1872). The expression of the emotions in man and animals. Communicating under Uncertainty.

[43] Arnold M.B. (1960). Emotion and Personality.

[44] Tomkins S.S. (1962). Affect, imagery, consciousness: Vol. I. The Positive Affects.

[45] Nabi R.L. (1999). A cognitive-functional model for the effects of discrete negative emotions on information processing, attitude change, and recall. Commun. Theory.

[46] Jiyeon S., Kai K., Hyunyi C. (2016). Reexamining Fear Appeal Models from Cognitive Appraisal Theory and Functional Emotion Theory Perspectives. Commun. Monogr..

[47] Dennis T.A., Cole P.M., Wiggins C.N., Cohen L.H., Zalewski M. (2009). The functional organization of preschool-age children’s emotion expressions and actions in challenging situations. Emotion.

[48] Frijda N.H. (1986). The Emotions.

[49] Bavel J.J.V., Baicker K., Boggio P.S., Capraro V., Cichocka A., Cikara M., Crockett M.J., Crum A.J., Douglas K.M., Druckman J.N. (2020). Using social and behavioural science to support COVID-19 pandemic response. Nat. Hum. Behav..

[50] Burges C.J.C. (1998). A tutorial on Support V ector Machines for pattern recognition. Data Min. Knowl. Discov. Vol..

[51] Haimson O.L. (2019). Mapping gender transition sentiment patterns via social media data: Toward decreasing transgender mental health disparities. J. Am. Med. Inform. Assoc..

[52] Mason C.H., Perreault W.D. (1991). Collinearity, power, and interpretation of multiple regression analysis. J. Mark. Res..

[53] Nabi R.L. (2003). Exploring the Framing Effects of Emotion: Do Discrete Emotions Differentially Influence Information Accessibility, Information Seeking, and Policy Preference?. Commun. Res..

[54] Memioglu T., Ozyasar M. (2022). Analysis of YouTube videos as a source of information for myocarditis during the COVID-19 pandemic. Clin. Res. Cardiol. Vol..

[55] Chan C., Sounderajah V., Daniels E., Acharya A., Clarke J., Yalamanchili S., Normahani P., Markar S., Ashrafian H. (2021). The Reliability and Quality of YouTube Videos as a Source of Public Health Information Regarding COVID-19 Vaccination: Cross-sectional Study. JMIR Public Health Surveill.

[56] Yoon S., McClean S.T., Chawla N., Kim J.K., Koopman J., Rosen C.C., Trougakos J.P., McCarthy J.M. (2021). Working through an “infodemic”: The impact of COVID-19 news consumption on employee uncertainty and work behaviors. J. Appl. Psychol..

[57] Hopwood T.L., Schutte N.S., Loi N.M. (2019). Anticipatory traumatic reaction: Outcomes arising from secondary exposure to disasters and large-scale threats. Assessment.

[58] Zillmann D. (2002). Exemplification Theory of Media Influence.

[59] Li Q., Wei C., Dang J., Cao L., Liu L. (2020). Tracking and Analyzing Public Emotion Evolutions During COVID-19: A Case Study from the Event-Driven Perspective on Microblogs. Int. J. Environ. Res. Public Health.

[60] Wang Y.W. (2020). Humor and Camera View on Mobile Short-Form Video Apps Influence User Experience and Technology-Adoption Intent, an Example of Tiktok (Douyin). Comput. Hum. Behav..

